# Novel *PAX9* Mutations Causing Isolated Oligodontia

**DOI:** 10.3390/jpm14020191

**Published:** 2024-02-08

**Authors:** Ye Ji Lee, Yejin Lee, Youn Jung Kim, Zang Hee Lee, Jung-Wook Kim

**Affiliations:** 1 Department of Pediatric Dentistry & DRI, School of Dentistry, Seoul National University, Seoul 03080, Republic of Korea; yeaji24@snu.ac.kr (Y.J.L.); thakdd0119@snu.ac.kr (Y.L.); ykim71@snu.ac.kr (Y.J.K.); 2 Department of Cell and Developmental Biology & DRI, School of Dentistry, Seoul National University, Seoul 03080, Republic of Korea; zang1959@snu.ac.kr; 3 Department of Molecular Genetics & DRI, School of Dentistry, Seoul National University, Seoul 03080, Republic of Korea

**Keywords:** hereditary, splicing mutation, oligodontia, *PAX9*, silent mutation

## Abstract

Hypodontia, i.e., missing one or more teeth, is a relatively common human disease; however, oligodontia, i.e., missing six or more teeth, excluding the third molars, is a rare congenital disorder. Many genes have been shown to cause oligodontia in non-syndromic or syndromic conditions. In this study, we identified two novel *PAX9* mutations in two non-syndromic oligodontia families. A mutational analysis identified a silent mutation (NM_006194.4: c.771G>A, p.(Gln257=)) in family 1 and a frameshift mutation caused by a single nucleotide duplication (c.637dup, p.(Asp213Glyfs*104)) in family 2. A minigene splicing assay revealed that the silent mutation resulted in aberrant pre-mRNA splicing instead of normal splicing. The altered splicing products are ones with an exon 4 deletion or using a cryptic 5’ splicing site in exon 4. Mutational effects were further investigated using protein expression, luciferase activity assay and immunolocalization. We believe this study will not only expand the mutational spectrum of *PAX9* mutations in oligodontia but also strengthen the diagnostic power related to the identified silent mutation.

## 1. Introduction

Human dentition is diphyodont with deciduous and permanent teeth, in contrast to its monophyodont nature in rodents and polyphyodont nature in toothed fishes and reptiles [1]. Human deciduous dentition consists of twenty teeth, five in each maxilla and mandible quadrants (two deciduous incisors, one deciduous canine and two deciduous molars), i.e., milk teeth. Human permanent dentition consists of twenty replacement successors (two incisors, one canine and two premolars) and eight molar teeth, excluding the third molars.

During early development, a series of reciprocal interactions between oral epithelium and cranial neural crest cells underlies tooth formation [2]. The entire tooth formation process is sequential, from bud to cap, to early bell and to late bell stages, and there are many molecular players involved in these complex processes. Environmental and/or genetic factors altering or damaging these processes could result in tooth agenesis or misshapen teeth such as peg lateralis or conical teeth.

Hypodontia, i.e., missing one or more teeth, is one of the most common congenital human diseases. The prevalence varies depending on the study populations and regions, but it was shown to be as low as 2.6% in a population study in Saudi Arabia and as high as 11.3% in a referral/clinical study in Ireland, both with the mandibular second premolar as the most affected tooth [3]. Therefore, hypodontia missing only one or two teeth does not belong to the category of rare disease. However, oligodontia missing six or more permanent teeth, excluding the third molars, is relatively rare, with a rate of 0.16% in Danish schoolchildren [4]. Anodontia, missing all teeth, is an extremely rare condition.

In this study, we recruited oligodontia families and performed genetic analysis to identify molecular genetic etiology. We used the candidate gene approach based on the genotype–phenotype relationship for family 1 and whole exome sequencing for family 2. We identified a silent mutation in family 1 and a frameshift mutation caused by single nucleotide insertion in family 2. Here, we report the mutational effect of both mutations and their molecular pathophysiology.

## 2. Materials and Methods

### 2.1. Patients’ Recruitment

This study and patients’ consent were independently reviewed and approved by the institutional review board of Seoul National University Dental Hospital (CRI05003G and 8 December 2022). The nature of this study was explained, and informed consent was obtained from all participants. Family histories were taken to draw the pedigree of each family. Clinical and radiological examinations were performed by the communicating author according to the principles in the Declaration of Helsinki.

### 2.2. Genomic DNA Isolation

Genomic DNA was isolated from 2 mL of peripheral blood by a conventional method with the NucleoSpin Blood L kit (Macherey-Nagel GmbH & Co., Düren, Germany) according to the manufacturer’s instructions. Candidate gene sequencing for the proband of family 1 was performed based on the genotype–phenotype relationship [5] using the following PCR primers (Table 1).

For family 2, the DNA samples of the proband and the affected mother were submitted for whole exome sequencing (BGI, Shenzhen, China). After shearing the genomic DNA with an ultrasonic high-performance sample processing system and adapter ligation, the whole exome was captured with the SureSelect Target Enrichment System (Agilent Technologies, Santa Clara, CA, USA) and amplified.

### 2.3. Bioinformatic Analysis

A series of bioinformatic analyses was used to process the obtained 100 bp paired sequencing reads, as previously described [6]. Briefly, after trimming the adapter sequences, the reads were aligned to the hg38 reference human genome assembly. Samtools and Genome Analysis Tool Kit were used to obtain a list of sequence variants, including single nucleotide changes and small insertions/deletions [7,8]. The dbSNP build 150 database was used for the reference to annotate the sequence variants, and the annotated variants were filtered with a minor allele frequency of 0.01.

### 2.4. Sanger Sequencing

The identified mutations and the segregation among the family members were confirmed by Sanger sequencing. PCR primers for exon 4 were used for the sequencing because both mutations were on exon 4 of the *PAX9* gene (NM_006194.4) (Table 1). Sanger sequencing was performed for all participating family members at the Macrogen sequencing center (Seoul, Republic of Korea). The identified *PAX9* mutations were submitted to the ClinVar database (https://www.ncbi.nlm.nih.gov/clinvar/ (accessed on 13 December 2023), Accession ID: SCV004175891 and SCV004175892).

### 2.5. In Vitro Splicing Assay

The wild-type and mutant genomic fragments, including exon 4 of the *PAX9* gene, were amplified from a DNA sample of the proband of family 1 using the Hifi PCR premix (Elpis Biotech, Daejeon, Republic of Korea) and cloned into the TOPcloner TA V2 vector (Enzynomics, Seoul, Republic of Korea) using the same primers as in the exon 4 sequencing. After the sequences were confirmed, the wild-type and mutant fragments were subcloned into the pSPL3 splicing vector after double digestion with the BamHI and XhoI restriction endonucleases. COS7 cells were transiently transfected with the wild-type and mutant pSPL3 vectors, total RNA was isolated after 36 h, and the cDNA was synthesized. Amplification bands from RT-PCR (SD6 sense primer 5′-TCTGAGTCACCTGGACAACC-3′ and SA2 antisense primer 5′-ATCTCAGTGGTATTTGTGAGC-3′) were excised from an agarose gel electrophoresis and characterized by direct DNA sequencing.

### 2.6. Wild-Type and Mutant PAX9 Expression Vectors

The PCR primer set [BglII_hPAX9-F (5′-agatctACCATGGAGCCAGCCTTCG-3′) and BamHI_hPAX9-R (5′-ggatcCTAGAGCGCGGAAGCCGTGAC-3′), amplicon size 1040 bp] was used to amplify the cDNA sequence in a clone from the DNASU plasmid depository (https://dnasu.org/DNASU/Home.do, accessed on 1 December 2022, clone ID: HsCD00817632). PCR was performed using the Pfu plus master mix (Elpis Biotech) and cloned into the TOPcloner blunt V2 vector (Enzynomics). Wild-type PAX9 expressing pEGFP vector was generated by subcloning using double digestion with the BglII and BamHI restriction endonucleases. PCR mutagenesis was performed to introduce mutations to be characterized (Table 2).

### 2.7. Transient Transfection and Western Blot

COS7 cells were transiently transfected with the wild-type and mutant PAX9 protein expression vectors using GenjetTM Ver. II (Signagen, Frederick, MD, USA) as the transfection reagent. After 6 h, the cell culture medium was changed to a complete medium. After 48 h post transfection, the cells were harvested, and the total proteins were extracted with the RIPA protein extraction solution (Elpis Biotech). Next, 50 µg of proteins were separated by 11% SDS-PAGE and transferred to nitrocellulose membranes (Schleicher & Schuell BioScience, Dassel, Germany). Membranes were blocked for 2 h with 5% skim milk in PBS and incubated overnight at 4 °C with primary antibody (PAX9; NBP3-10316, Novus Biologicals, Centennial, CO, USA, and GAPDH; G041, abm Inc., Richmond, Ontario, Canada) diluted in PBS-T buffer (1:10,000). After washing, the membranes were incubated for 1.5 h with secondary antibodies (#31460, Thermo Fisher Scientific, Rockford, IL, USA, and G21040, Invitrogen, Thermo Fisher Scientific). Labeled protein bands were detected with a PicoEPD Western Reagent (Elpis Biotech) and X-ray autoradiography system.

### 2.8. Luciferase Reporter Assay

COS7 cells were transfected with the wild-type and mutant PAX9 protein expression vectors with pAM5 and pRL-TK (Promega, Madison, WI, USA). The pAM5 is a reporter plasmid that the SV40 early promoter of the pGL4.13 luciferase reporter vector is replaced with a 2.3 kb *BMP4* promoter fragment (a generous and kind gift from Dr. Shinji Yashuhira, Iwate Medical University) [9]. The luciferase activity was measured using a dual-luciferase reporter assay system (Promega) according to the manufacturer’s instructions with a multi-mode microplate reader (Synergy H1, Agilent Bio-Tek, Thermo Fisher Scientific).

### 2.9. Immunocytochemistry

COS7 cells transfected with the wild-type and mutant PAX9 protein expression vectors were fixed and permeabilized as described before [10]. Cells were treated with the PAX9 primary antibody in a dilution of 1:500 at 4 °C overnight. After washing with 1% BSA in 1× PBS for 10 min 3 times, goat anti-rabbit IgG antibody (H+L) Texas Red (TI-1000, Vector Laboratories, Inc., Burlingame, CA, USA) was used at a titer of 1:500 and incubated for 1.5 h at room temperature. Fluoroshield with DAPI (Sigma-Aldrich, St Louis Mo, USA) was used to stain nucleic acid, and the cells were examined by confocal laser scanning microscopy (LSM700, Carl Zeiss, Oberkochen, Germany) at 630× magnification.

## 3. Results

### 3.1. Family 1

**Figure 1:**
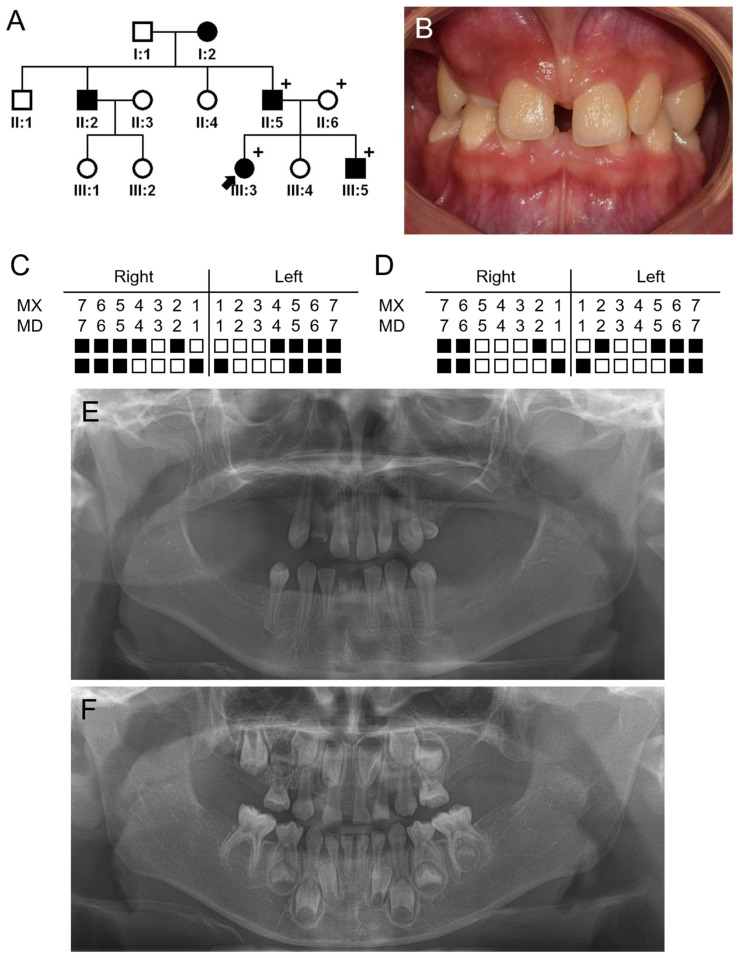
Pedigree, clinical photo and panoramic radiographs of family 1. (**A**) Pedigree of family 1. Black symbols indicate affected individuals, and the proband is indicated by a black arrow. Plus, signs above the symbols indicate participating individuals. (**B**) Clinical photo of the proband at age of 11 years and 7 months. (**C**) Summary chart of missing teeth of the proband. She is missing 17 permanent teeth (black-filled symbol means missing tooth). (**D**) Summary chart of missing teeth of the affected sibling (III:5). He is missing 13 permanent teeth and 3 deciduous teeth (maxillary right and left second deciduous molars and maxillary right lateral deciduous incisor). (**E**) Panoramic radiograph of the proband at age of 11 years and 7 months. (**F**) Panoramic radiograph of the affected sibling (III:5) at age of 5 years and 4 months.

The proband of family 1 was an 11-year-7-month-old girl from a nonconsanguineous Korean family. She was the oldest among the three siblings, and her 5-year-4-month-old brother was also affected with oligodontia (Figure 1). She was missing 17 permanent teeth, and her brother was missing 13 permanent teeth and 3 deciduous teeth. However, there was no other remarkable past medical history and developmental alterations, including but not limited to those related to the hair, sweat glands and nails. This condition was inherited from her father and grandmother. According to the pedigree, an autosomal dominant inheritance pattern was highly suspected.

**Figure 2:**
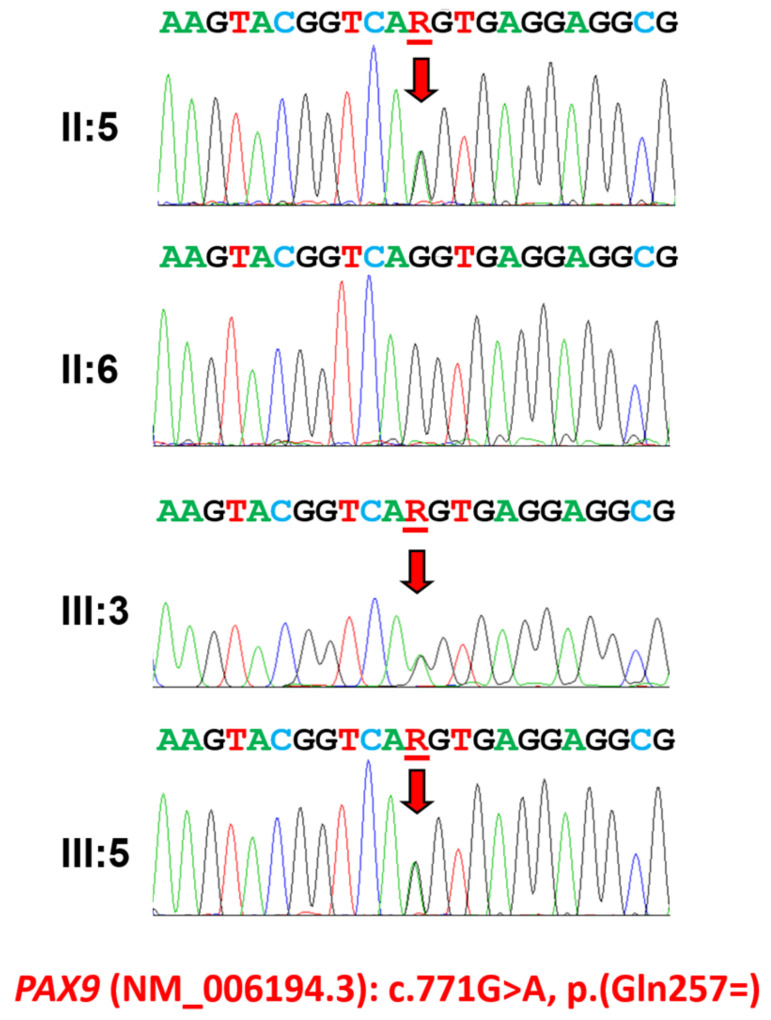
Sequencing chromatograms of family 1. Sequencing chromatograms of the participating individuals of family 1. Nucleotide sequences are shown above the chromatograms. Nucleotide affected by the mutation is underlined and indicated with a red arrow (NM_006194.4: c.771G>A). Individual identifications are indicated on the left side of each chromatogram.

In addition to the inheritance pattern, phenotype features such as locations of missing teeth (posterior teeth missing), no alteration in the other tissues and consistent phenotypes among affected family members suggested the *PAX9* gene as a primary candidate target. Candidate gene sequencing revealed a transition mutation changing the last nucleotide of exon 4 from a guanine to an adenine (NM_006194.4: c.771G>A) (Figure 2). The mutation was never reported in any of the databases, including the dbSNP, 1000 genomes and gnomAD database (https://gnomad.broadinstitute.org/, accessed on 1 December 2023), and segregates perfectly with the disease in the family; however, the mutation does not change the amino acid encoding; therefore, it is a silent mutation (NP_006185.1: p.(Gln257=)).

**Figure 3:**
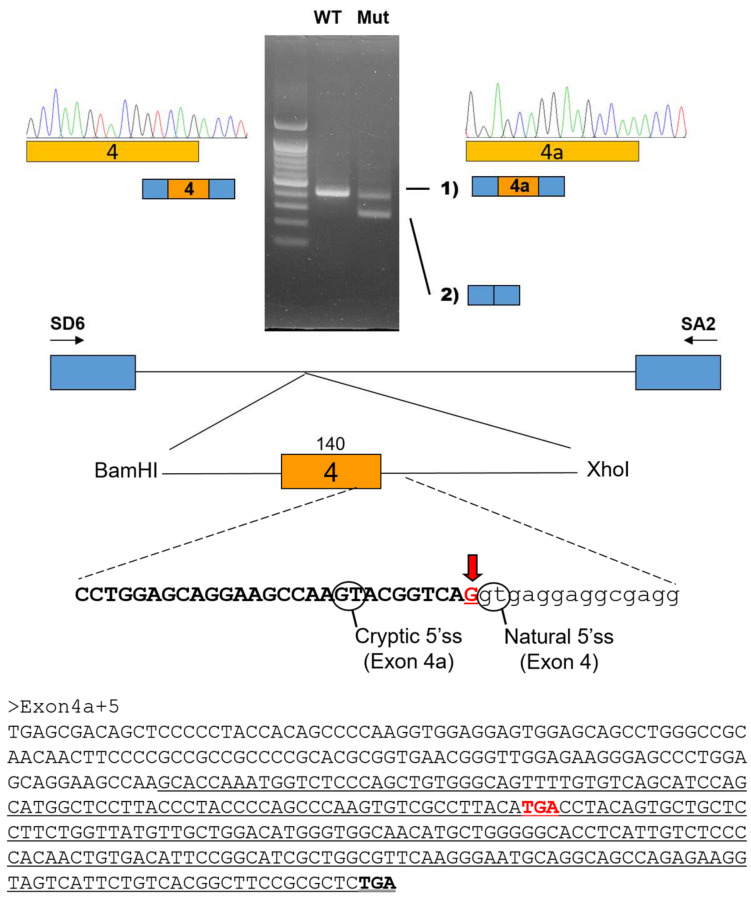
In vitro splicing assay. A genomic fragment, including exon 4 of the *PAX9* gene, was subcloned into the pSPL3 vector with double digestion using the BamHI and XhoI restriction endonucleases. Boxes indicate exons, and horizontal lines indicate introns. The number of the exon is in the box, and the length of the exon is shown above the box. Locations of the primer binding site are indicated with arrows (sense and antisense). Agarose gel image of the splicing assay of the wild type (WT) and mutant (Mut) is shown on the top. Left lane is the DNA ladder. Wild-type vector resulted in a normal splicing product, including the entire exon 4 shown on the left with the sequencing chromatogram. Mutation (c.771G>A) resulted in an exon 4 deletion (x4del) or an altered transcript with a cryptic 5’ splicing site in exon 4 (x4a). Sequencing chromatogram of the alternative splicing band is shown on the right. The mRNA sequence of exon 4a and 5 is shown below. The black-bolded TGA is the normal translation termination codon, and the red-bolded TGA is the premature stop codon. The exon 5 sequences are underlined.

The minigene splicing assay revealed that the silent mutation affected normal splicing (Figure 3). The mutation resulted in two altered spliced transcripts instead of the normally spliced transcript: one with an exon 4 deletion (x4del) and one with a cryptic 5’ splicing site in the exon 4 (x4a). The x4del mutant transcript skips the entire 140 bp exon 4, resulting in a frameshift and premature stop codon in exon 5 (c.632_771del, p.(Val211Glyfs59*)). The location of the premature stop codon in the last exon would enable escaping from the nonsense-mediated mRNA decay surveillance system (NMDS); therefore, the mutant transcript is predicted to produce a 268-amino-acid-truncated protein instead of the 341-amino-acid wild-type PAX9 protein. The other mutant transcript, x4a, uses a cryptic 5’ splicing site 10 nucleotides ahead of the normal splicing site, therefore resulting in a 10 bp frameshift deletion (c.762_771del, p.(Tyr255Hisfs*30)). The frameshift also introduces a premature stop codon in the last exon and, therefore, would produce a 283-amino-acid-truncated protein.

### 3.2. Family 2

**Figure 4:**
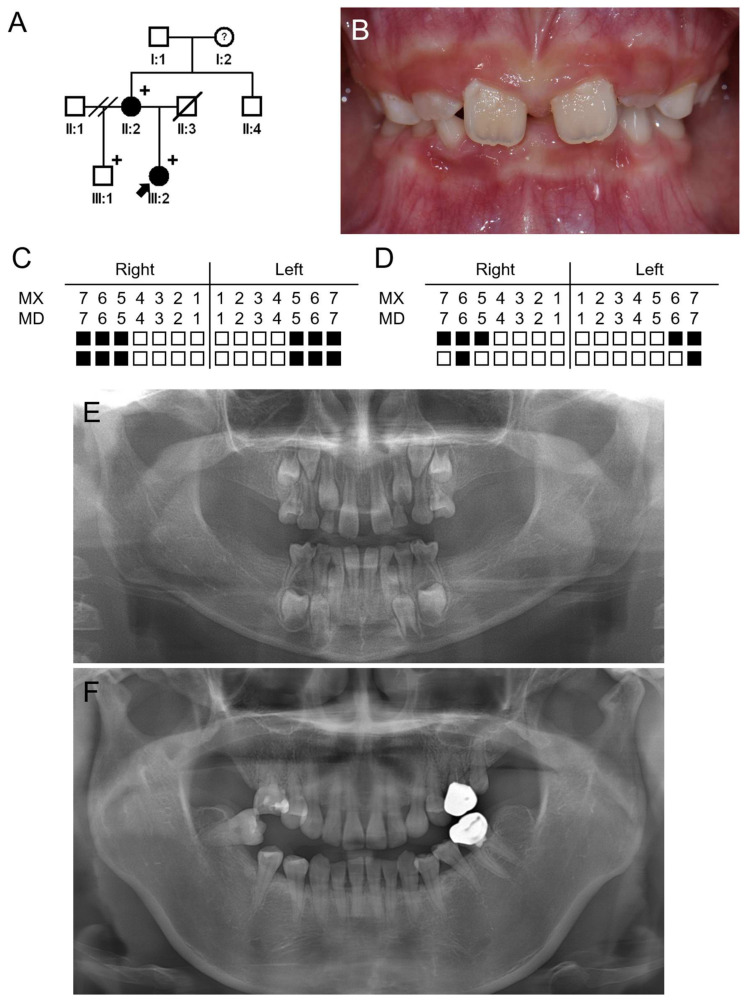
Pedigree, clinical photo and panoramic radiographs of family 2. (**A**) Pedigree of family 2. Black symbols indicate affected individuals, and the proband is indicated by a black arrow. Plus, signs above the symbols indicate participating individuals. A question mark in the symbol means unclear phenotype. (**B**) Clinical photo of the proband at age of 6 years and 1 month. (**C**) Summary chart of missing teeth of the proband. She is missing 12 permanent teeth and 4 deciduous teeth (all second deciduous molars). (**D**) Summary chart of missing teeth of the affected mother (II:2). She is missing 7 permanent teeth. (**E**) Panoramic radiograph of the proband at age of 6 years and 1 month. (**F**) Panoramic radiograph of the affected mother (II:2) at age 41 years.

The proband of family 2 was a 6-year-1-month-old girl from a nonconsanguineous Korean family (Figure 4). She was a child from the second marriage of her mother and was missing 12 permanent teeth and 4 deciduous teeth (all second deciduous molars). She has no remarkable past medical history, and there were no symptoms related to ectodermal dysplasia in the hair, skin and nails. Her father died due to gastric cancer but did not have any missing teeth. Her 41-year-old mother had no other medical symptoms except oligodontia, missing seven permanent teeth. Her grandfather has no missing teeth. Her grandmother is currently wearing dentures, but it is not clear whether she is missing teeth, and it was said that several teeth fell out due to trauma.

**Figure 5:**
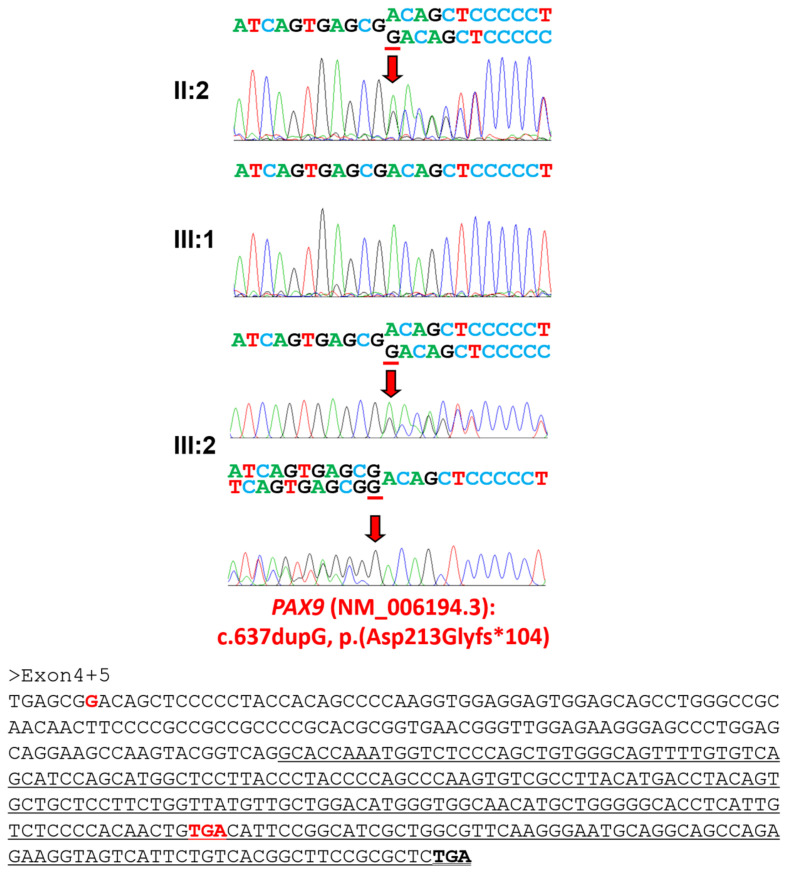
Sequencing chromatograms of family 2. Sequencing chromatograms of the participating individuals of family 2. Nucleotide sequences are shown above the chromatograms. Upper chromatogram of the proband is forward sequencing, and the lower one is reverse sequencing. Nucleotide affected by the mutation is underlined and indicated with a red arrow (NM_006194.4: c.637dup). Individual identifications are indicated on the left side of each chromatogram. The mRNA sequences of exons 4 and 5 are shown below. The black-bolded TGA is the normal translation termination codon, and the red-bolded TGA is the premature stop codon. The exon 5 sequences are underlined.

The whole exome sequencing of the proband and her mother resulted in about x100 median target coverage with more than 96% coverage (Table 3), and the analysis revealed that only one mutation was shared among the genes causing tooth agenesis. The mutation was a duplication of a guanine causing a frameshift (NM_006194.4: c.637dup) (Figure 5), and the frameshift introduces a premature stop codon in the last exon that would produce a 315-amino-acid-truncated protein (NP_006185.1: p.(Asp213Glyfs*104)).

### 3.3. Luciferase Assay, Western Blot and Immunocytochemistry

**Figure 6:**
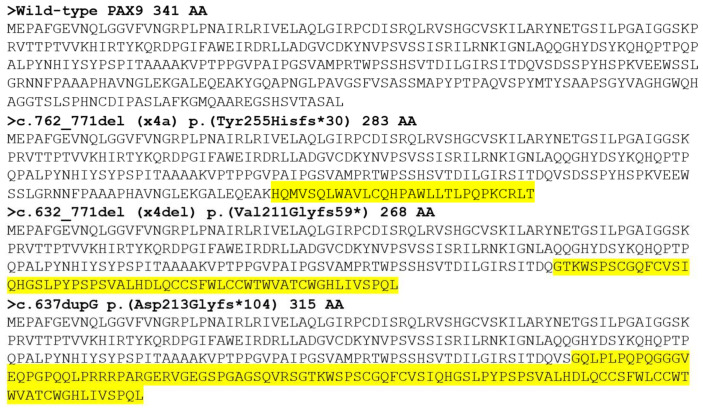
Amino acid sequences of the wild-type and mutant PAX9 proteins. Mutations in cDNA and protein are shown above the sequences. The length of the amino acids is shown, and the novel sequences are highlighted in yellow.

**Figure 7:**
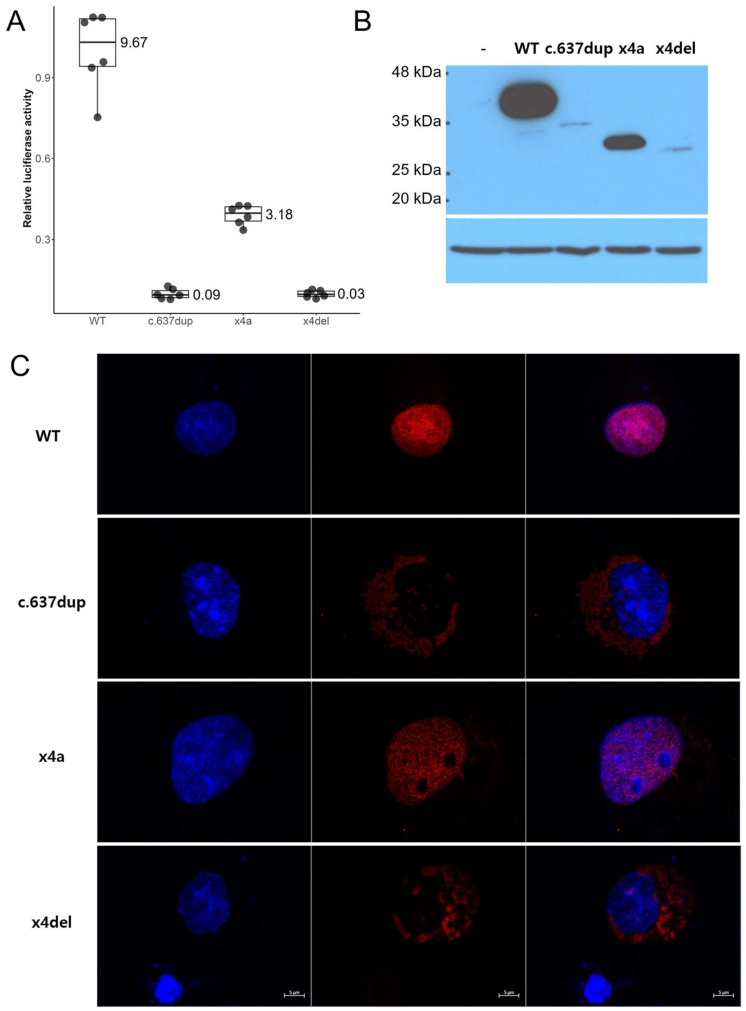
Luciferase assay, Western blot and immunocytochemistry. (**A**) Relative luciferase activity of wild type (WT), c.637dup, c.762_771del (x4a) and c.632_771del (x4del). Mean values are shown on the right of the boxes. (**B**) Western blot. Expressions of c.637dup and x4del were extremely low, and expression of x4a was reduced compared to the wild type. - means negative control with empty pEGFP vector. (**C**) Immunocytochemical analysis revealed that x4a was localized to the nucleus like wild type, but c.637dup and x4del cannot enter the nucleus. Blue color (left panel) is DAPI stain, and red color (middle panel) is Texas Red for PAX9. Right panel is a composite. Length bar of 5 µm is shown on the right below.

Wild-type and mutant PAX9 expression cDNA clones were used to test the luciferase activity, expression and localization (Figure 6). The relative luciferase activity of the c.637dup and c.632_771del (x4del) were extremely weak (mean 0.09 and 0.03). Even though the c.762_771del (x4a) showed some luciferase activity (mean 3.18), it was much less compared to the wild type (mean 9.67) (Figure 7A). Western blot showed a similar pattern; the expression level of x4a was moderately reduced compared to the wild type, but the expressions of c.637dup and x4del were greatly reduced (Figure 7B). Immunocytochemistry showed that the c.637dup and x4del localized in the cytoplasm, while the wild type and x4a localized in the nucleus (Figure 7C).

## 4. Discussion

Among the genes causing non-syndromic oligodontia, mutations in the *PAX9* gene have unique phenotypic characteristics. Posterior teeth are preferentially affected more than any other oligodontia cases caused by other genes. More than 80% of maxillary and mandibular second and third molars are missing in most cases. The first molars are also frequently missing, especially in the maxilla (69~79%) compared to the mandible (32~44%) [5,11,12,13,14].

PAX9 and MSX1 are indispensable transcription factors involved in tooth development [15,16]. They share a similar expression pattern, especially in the mesenchymal cells predominantly, and the knockout mice of each factor had an arrest in the transition from bud to cap stages in homozygotes, while there is no specific phenotype in heterozygotes [17,18]. Additionally, the homozygotes died within a day, and they also had other characteristics, such as cleft secondary palate, deficiency of alveolar bone and disturbed craniofacial skeletogenesis. However, human phenotypes are rather different, even between the two transcription factors. Heterozygous mutations in the *MSX1* or *PAX9* gene cause oligodontia; therefore, they share an autosomal dominant inheritance in humans [19,20]. It seems that humans have less tolerance to the reduction in or loss of functional regulation by these transcription factors in tooth development. *MSX1* mutations sometimes exhibit other syndromic phenotypes, such as the variable expression of a cleft palate only or cleft lip and palates [21] and nail dysgenesis (Witkop syndrome) [22]. In addition to the permanent teeth missing, missing primary teeth have been reported in some cases with *PAX9* mutations [23,24,25] but not with *MSX1* mutations [5]. Missing primary teeth were also seen in both families in this study.

In this study, we identified two novel *PAX9* mutations causing non-syndromic oligodontia. The identified mutation in family 1 was a nucleotide change in the last nucleotide of exon 4 which does not change the amino acid encoding (NM_006194.4: c.771G>A; CAG to CAA: both encoding a glutamine). However, it turned out that this silent mutation abolished a canonical 5’ splicing donor site in intron 4 and resulted in altered mRNAs with an exon 4 deletion (x4del) and a cryptic 5’ splicing site in exon 4 (x4a). Both altered mRNA transcripts were predicted to escape NMDS and encode truncated PAX9 mutants (x4del: c.632_771del, p.(Val211Glyfs59*) and x4a: c.762_771del, p.(Tyr255Hisfs*30)). The identified mutation in family 2 was a frameshift mutation caused by the duplication of a guanine, and this mutation was also predicted to escape NMDS and encode a truncated PAX9 protein (c.637dup, p.(Asp213Glyfs*104)).

However, an in vitro test revealed that the expressions of x4a and c.637dup were reduced almost to nothing, probably due to the endoplasmic reticulum quality control system for misfolded proteins [26]. Furthermore, these weakly expressed proteins could not enter the nucleus; thus, their transcriptional activity was not expected, as seen in the luciferase assay. The x4a mRNA utilizing a cryptic splicing site, however, was expressed in a moderately reduced level and still retained nucleus localization. The relative luciferase assay showed about 1/3 of the wild-type activity.

## 5. Conclusions

In conclusion, we identified novel *PAX9* mutations causing non-syndromic oligodontia through candidate gene sequencing or whole exome sequencing. We characterized the mutational effects on mRNA splicing, protein expression and immunolocalization. This study will not only expand the mutational spectrum of the *PAX9* mutations in oligodontia but also improve diagnostic confidence related to the identified silent mutation.

## Figures and Tables

**Table 1:** PCR primers for the *PAX9* gene.

Exons	Forward Primer	Reverse Primer	Amplicon Size
Exon 2	ACCAGCCTGATTTTGCTGTC	AGAATGTGAGCGCCTAGTGG	584
Exon 3	CGCGCTGTGTGTTCATTTT	AGACGCTGCACATCCACAC	690
Exon 4	TGGAAAGGCCTACTCTGAGG	GAAGGATCTGGCTCGTAGCA	499
Exon 5	TCAGAGCATTGCTGGCTTAC	CTTTCAAGGCAGAAGGGTTG	481

Exon numbers are based on the reference sequence for mRNA (NM_006194.4).

**Table 2:** Primers for PCR mutagenesis.

Names	Forward Primer	Reverse Primer
hPAX9_c.637dup	CAAGTGAGCGGACAGCTCCCC	GGGGAGCTGTCCGCTCACTTG
hPAX9_X4a	CAGGAAGCCAAGCACCAAATGG	CCATTTGGTGCTTGGCTTCCTG
hPAX9_X4del	CACCGACCAAGGCACCAAATGGTCTC	GAGACCATTTGGTGCCTTGGTCGGTG

**Table 3:** Statistics for exome sequencing.

Sample	Total Reads	Mapping Rate (%)	Median Target Coverage	Coverage of Target Region (%)	Fraction of Target Covered with at Least	
					20×	10×
Family 2 II:2	126,220,376	99.9	101	96.2	94.4	95.5
Family 2 III:2	128,766,878	99.9	108	96.3	94.6	95.6

## Data Availability

The data presented in this study are openly available in ClinVar (http://www.ncbi.nlm.nih.gov/clinvar/, accessed on 13 December 2023), Accession ID: SCV004175891 and SCV004175892.
